# Usefulness of ectopic implantation in multiple finger amputation injury

**DOI:** 10.1002/ccr3.2040

**Published:** 2019-02-07

**Authors:** Shuhei Yoshida, Isao Koshima, Mitsunaga Narushima, Shogo Nagamatsu, Kazunori Yokota, Shuji Yamashita, Mitsunobu Harima

**Affiliations:** ^1^ International Center for Lymphedema (ICL) Hiroshima Japan; ^2^ Department of Plastic and Reconstructive Surgery Mie University Mie Japan; ^3^ Plastic and Reconstructive Surgery Hiroshima University Hospital Hiroshima Japan; ^4^ Department of Plastic and Reconstructive Surgery Graduate School of Medicine The University of Tokyo Tokyo Japan

**Keywords:** ectopic implantation, microsurgery, multiple finger amputation, team surgery

## Abstract

Replantation combined with simultaneous ectopic implantation can be considered in patients with multiple finger amputation injury. This technique has the advantages of reducing the operating time and optimizing hemodynamic stability. However, it is only possible when multiple hand and microsurgery team can be organized at short notice.

## 1 INTRODUCTION

Replantation of multiple amputated fingers is generally performed one finger at a time depending on the extent of damage because of the limited operative field that can only be viewed through a microscope. It is possible to reduce the burden on the operator by taking turns if multiple microsurgical teams can be organized. However, it is still impossible, even for a large microsurgery team, to reduce the operating time and the physical burden on the patient when replanting fingers one by one because of the restricted operating field. We attempted to shorten the operating time by performing conventional one‐by‐one replantation and ectopic implantation simultaneously and examined the usefulness of this strategy.

## 2 METHOD

**Figure 1:**
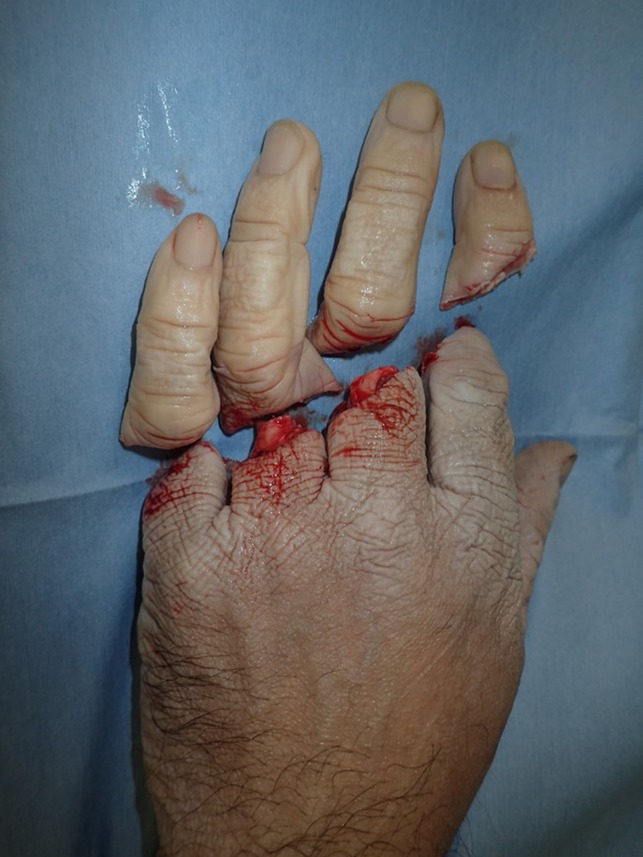
The patient was a 44‐year‐old man with a history of myocardial infarction who sustained a four‐finger amputation injury involving the index to little fingers of his left hand while using a rubber cutting machine

The patient was a 44‐year‐old man with a history of myocardial infarction who sustained a four‐finger amputation injury involving the index to little fingers of his left hand while using a rubber cutting machine. He was brought to our hospital with the four amputated fingers. The amputations were clear‐cut and at the level of the middle phalanx in the index finger and the proximal phalanx in the middle, ring, and little fingers. Cold ischemia time was 240 minutes on arrival at the operating room (Figure 1).

Despite the operation needing to be performed on an emergency basis, it was possible to organize two teams. In view of the patient's past medical history, we decided to perform simultaneous ectopic implantation in parallel with the conventional one‐by‐one replantation to shorten the operating time, reduce the physical burden on the patient, and allow for unexpected intraoperative events.

The first team started replantation of the index finger to the native position while the second team started ectopic transfer of the middle finger to the right thigh using the descending branch of the lateral circumflex femoral artery as the recipient vessel.

Replantation to the native position was performed with C‐wire bone fixation, suturing of the flexor and extensor tendons, and anastomosing two digital nerves and arteries and two subdermal veins on the dorsal side in all fingers.

**Figure 2:**
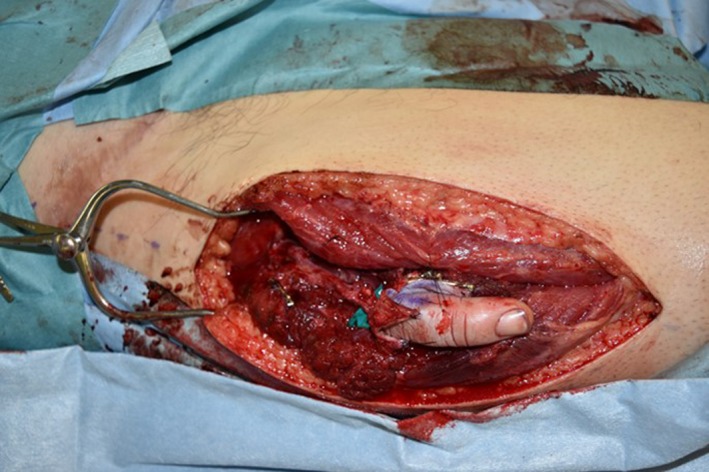
While replanting the index finger to its native position, the second team dissected the descending branches of the lateral circumflex femoral artery. The ectopic implantation was performed to middle finger

While replanting the index finger to its native position, the second team dissected the descending branches of the lateral circumflex femoral artery. The ectopic implantation was performed by anastomosing two digital arteries to the descending branches of the lateral circumflex femoral artery and two subdermal veins on the dorsal side of finger to two collateral veins of the descending branch of the lateral circumflex femoral veins (Figure 2). The diameters of the vessels were closely matched between the amputated finger and the recipient site. While the second team was anastomosing the middle finger, the first team replanted the little finger to its native position in the same manner as that used for the index finger. Although 6 hours of operating time had passed by this time, the patient's general condition was still stable. Therefore, we decided to continue the surgery and replant his ring finger to its native position.

**Figure 3:**
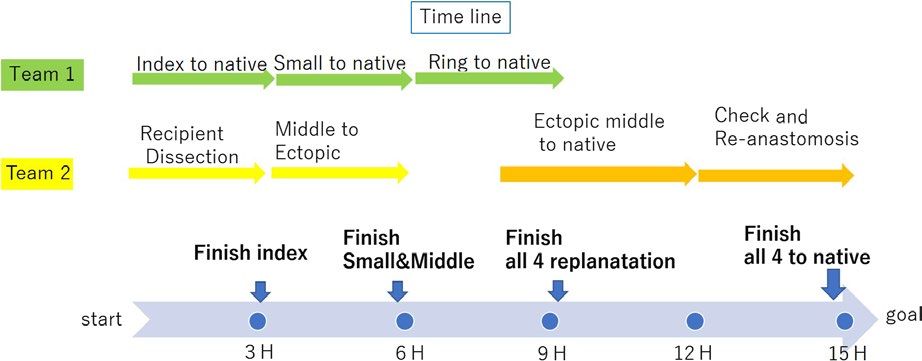
Nine hours had passed by the time we finished replanting all four fingers. The final operating time was 15 h to complete all four replantations to their native positions

Nine hours had passed by the time we finished replanting all four fingers. The patient's general condition continued to remain stable, so we took the decision to complete all four replantations to their native positions, which involved re‐replanting the middle finger to its native position from the initial ectopic replantation position in the hope of improving its function postoperatively. We harvested the descending branches of the lateral circumflex femoral artery and collateral veins as conduits for transfer to the amputation site with bone fixation by C‐wire and suturing of the flexor and extensor tendons. The final operating time was 15 hours, during which time the patient remained in a stable condition (Figure 3).

## 3 RESULTS

**Figure 4:**
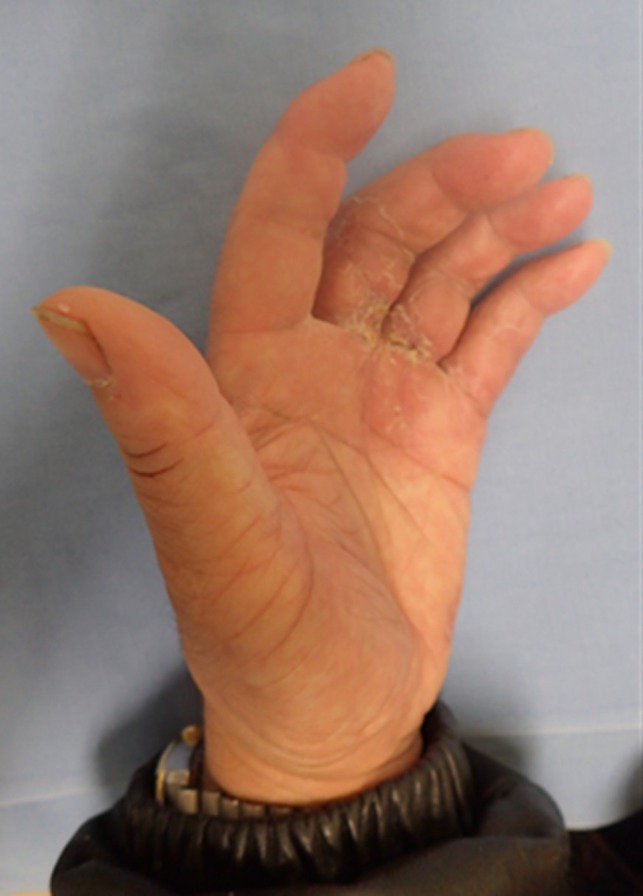
All the fingers have survived. The patient reports slight stiffness in the replanted fingers but no problems in performing normal activities of daily living

The postoperative course was uneventful, and circulation was stable in all fingers. As of 2 years after the procedure, all the fingers have survived. The patient reports slight stiffness in the replanted fingers but no problems in performing normal activities of daily living (Figure 4).

## 4 DISCUSSION

The first successful clinical application of ectopic implantation of a body part was reported in 1984.^1^ Since then, there has been a growing body of reports describing use of this technique for temporary banking of amputated body parts.^2^, ^3^, ^4^, ^5^, ^6^, ^7^, ^8^, ^9^, ^10^, ^11^, ^12^, ^13^, ^14^, ^15^ This creative and useful concept serves as an important limb‐saving technique for the reconstructive hand surgeon in selected clinical settings.

This surgical technique has been suggested for patients in whom there is extensive soft tissue loss or contamination, and the amount of radical debridement required to allow immediate replantation would result in extensive shortening of the amputation stump and debridement of vital structures. Moreover, it has been emphasized that the main indication for ectopic banking is to obviate the need for excessive shortening at the time of injury and allow preservation of limb length via open wound care and coverage before delayed replantation of the amputated body part. However, the recommendations regarding the surgical indications, ideal location and duration of ectopic banking, method and timing of management of soft tissue, and management of complex microvascular secondary replantation are variable^16^.

Variations in this technique include harvesting of a “free fillet flap” from the amputated limb and banking it for reconstruction of the stump,^4^, ^17^, ^18^”crossover replantation” in the case of bilateral amputations whereby a superior amputated extremity is replanted to the better contralateral stump,^5^, ^19^ and harvesting of a “prefabricated chimeric flap” by raising an ectopically implanted digit with a perforator flap and a vascularized nerve with a sufficiently long pedicle as a chimeric flap for second‐stage reconstruction^20^ and reattachment of an amputated penis^15^ or scalp tissue.^14^

Unlike with conventional ectopic transfers, the plan for our patient was to shorten the operating time and reduce the physical burden on the patient because of his past history of myocardial infarction. Mindful of the possibility of unexpected intraoperative difficulties, we elected to perform ectopic implantation in parallel with conventional one‐by‐one replantation of the severed fingers. However, the patient's intraoperative condition remained stable, so we decided to re‐replant the middle finger to the native position after ectopic implantation during the initial replantation surgery in the hope of improving postoperative function, as in previous reports.^2^, ^6^, ^9^, ^11^, ^16^, ^17^ All the replantations were completed in around 15 hours. However, we estimate that combined replantation with ectopic implantation could have decreased the operating time to around 9 hours (ie, to 60% of that required for standard replantation), given that ectopic implantation can be performed in a relatively short time and requiring only vascular reconstruction without the tendon, bone, and nerve work necessary in standard replantation.^7^ Another advantage of ectopic transfer in amputation injuries involving multiple fingers is that replantation can be performed in both ectopic and native positions under a wider operative field because there is no interference from adjacent swollen fingers. As described elsewhere, the technique is particularly helpful for achieving hemodynamic stability, in that the entire lengths of the ectopic recipient artery and vein can be harvested as conduits for transfer to the amputated body part.

The main limitation of this technique is that it can only be performed in facilities that can organize more than two hand and microsurgery teams at short notice. Furthermore, although the operating time and burden of the initial surgery to both the surgical team and patient may be shortened, the need for a second operation would increase the burden on the patient. However, this could be minimized by making arrangements for appropriate examinations and planning the second operation well in advance. All the replanted fingers survived in our patient. He reports some slight stiffness in the replanted fingers but no problems in activities of daily living. Therefore, this combined strategy could be one of the surgical treatment options in patients with multiple finger amputation injuries.

Finally, it should be noted that we selected the descending branches of the lateral circumflex femoral artery as the recipient vessels for ectopic implantation, which is not common when using this technique. However, we chose these vessels because it was preferable to perform multiple ectopic implantations using only one recipient vessel, the diameter of the vessels of the amputated fingers was estimated to be around 1 mm, and use of only one vessel would not affect the patient's ability to mobilize postoperatively.

## 5 CONCLUSION

Replantation combined with simultaneous ectopic implantation can be considered in patients with multiple finger amputation injury. This technique has the advantages of reducing the operating time and optimizing hemodynamic stability. However, it is only possible when multiple hand and microsurgery team can be organized at short notice.

## CONFLICTS OF INTEREST

None of the authors has a financial interest in any of the products, devices, or drugs mentioned in this manuscript.

## AUTHOR CONTRIBUTION

YoS: performed as main surgeon of the work, writing and revising the article, and involved in management of all the work. KI: participated in management of the patient, drafting the article, critical revision, and final approval of the version to be published. NM: performed as main surgeon of the work and contributed to the concept. YaS: participated in management of the patient. HM: participated in management of the patient. NS: participated in drafting the article, critical revision, and final approval of the version to be published. YK: participated in drafting the article, critical revision, and final approval of the version to be published.
